# The County Council's New Scheme Explained

**Published:** 1914-03-14

**Authors:** 


### A LONDON AMBULANCE SERVICE.

### The County Council's New Scheme Explained.

The London County Council's scheme for providing an ambulance service for London is now public property, the Report of the General Purposes Committee having been passed by the Council at its meeting on Tuesday, March 10. The steps leading up to the preparation of the present scheme have been alluded to in The Hospital on many occasions. It is sufficient here to say that after the rejection of the Asylums Board's scheme a special sub-committee of the General Purposes Committee was appointed to go into the whole matter, and the proposal now submitted to the Council is the result of their deliberations.

### A Tentative Project.

The first striking feature in the scheme is its low cost. The General Purposes Committee put the expenditure for maintenance at from £9,000 to £10,500 a year, but they are careful to add that the tentative scheme now brought forward, although they believe it will meet to a great extent the present need, will, in the future, need considerable enlargement. Existing appliances can be used only as supplemental to the service.

Six stations are to be provided—three north and three south of the Thames—each of which will be equipped with one motor ambulance, except the Shoreditch Station, which will be provided with two ambulances. The Central Ambulance Station, through which all calls will be transmitted, will be situated temporarily at the head-quarters of the Fire Brigade in Southwark Bridge Road. This station will have as many direct telephonic communications with exchanges as may be found desirable, and also, as the scheme develops, probably with some of the larger general hospitals. The six ambulance stations equipped with motor ambulances will be established as follows: North of the Thames—North End Road, Fulham (disused fire station); Devonshire Mews, Harley Street (building to be rented); and Boundary Street Estate, Shoreditch (the property of the Council); while those south of the Thames will be—London Road, Southwark; Lee High Road (opposite Belmont Park); and a site at Brixton Hill near the Lambeth Town Hall. Where possible, existing buildings will be utilised, and where buildings have to be provided temporary iron buildings have been selected. From these stations it is believed that all parts of the county can be served, and future extensions or alterations can be made as experience directs. It is intended that calls for an ambulance shall be made by the London telephone system to the Central Station, from which the call will be transmitted direct to the ambulance station nearest to the site of the accident. The provision of special call-boxes has been rejected as unnecessary. In Central London no difficulty as to telephones is likely to arise, but in the outlying districts they are not so numerous, but it is suggested that small notification plates should be attached to indicate where the telephone may be used by a constable in uniform. The service will be available for use for street accidents and street illness and for cases of accident in factories and private houses, but not for the conveyance of sick persons from private houses. The motor ambulance at Hampstead General Hospital, which is already available by arrangement with the Council for street accident cases, will be used as an adjunct to the service, and will, if necessary, be placed in direct communication with the Central Station, as will also be any other rapid ambulances belonging to public authorities which may be placed at the disposal of the Council for use in cases of emergency.

### The Staff.

As it is felt that there are many points of resemblance, it is proposed that the service shall be controlled by the chief officer of the Fire Brigade, but the advice of the medical officer of health will be available on all questions relating to the medical side of the service. While the direction of the service is to be entrusted to the Fire Brigade Committee, it is intended that the ambulance service shall be regarded as separate from the Fire Brigade. At as early a date as possible an officer is to be appointed at a salary of £400 a year, rising to £500 a year, who will be directly responsible to the chief officer of the Fire Brigade for the discipline and the effective working of the service. A telephone operative staff will be installed at the Central Station, the supervisor residing on the premises, and at each station there will be drivers and ambulance attendants. It has not yet been decided whether one or two attendants, in addition to the driver, shall accompany the ambulance, as it depends whether a constable summoned to a street accident will accompany the injured person to the hospital. One day's rest in seven will be given to each man, and the rate of wages, which has not been fixed, will depend on whether the men are fully trained on entering or whether they have to be specially trained after engagement.

The adoption of petrol-driven vehicles is recommended. The bodies of the ambulances will be arranged to receive two patients in a prone position, and will be well ventilated, the interior being perfectly smooth and without sharp corners where dust could accumulate. It has been decided to adopt the dhooly type of stretcher, with the addition of an attachment to make the stretcher rigid when necessary.

### The Financial Aspect.

The General Purposes Committee confess that they are not yet in a position to give detailed estimates. In order that the motor ambulances shall be ordered as soon as possible, an estimate of £5,400 is put forward to cover their cost, and each vehicle will be placed on duty immediately upon delivery. The expenditure on such items as adaptations of buildings for stations, provision of temporary buildings, furniture, stores, medical fittings, and training of staff (if necessary) is put at £2,000. The expenditure for rent, rates, taxes, depreciation, maintenance, stores, and wages is estimated at from £9,000 to £10,500 a year. The Finance Committee consider that on this estimate the cost for each of the seven working ambulances is high.

From statistics prepared by the Commissioner of Police it appears that in about 50 per cent. of the cases of injury caused by vehicular traffic the police convey the sufferers to the hospital. Consequently, of 19,000 such cases in 1913 in the county of London, 9,500 were conveyed to the hospital by the police; and of 5,250 cases of serious illness in the street, about 5,000 were conveyed by the police to hospitals. From the information given in the Report of the Departmental Committee, the total number of such cases actually conveyed to hospitals from the street is put at 20,700. It is estimated that at least another 7,000 cases from factories and workshops will have to be provided for, making a total of 27,700. The General Purposes Committee add that, having regard to the very marked increase of street cases, the service will eventually be one of considerable magnitude.

After a long debate on Tuesday the proposals were unanimously adopted as the basis of a tentative scheme. During the discussion Mr. Edward Smith referred with appreciation to the efforts of The Hospital to interest public opinion in the matter.

